# Opportunistic feeding behaviour and *Leishmania infantum* detection in *Phlebotomus perniciosus* females collected in the human leishmaniasis focus of Madrid, Spain (2012–2018)

**DOI:** 10.1371/journal.pntd.0009240

**Published:** 2021-03-15

**Authors:** Estela González, Ricardo Molina, Andrés Iriso, Sonia Ruiz, Irene Aldea, Ana Tello, Daniel Fernández, Maribel Jiménez

**Affiliations:** 1 Laboratorio de Entomología Médica, Centro Nacional de Microbiología, Instituto de Salud Carlos III, Majadahonda, Madrid; 2 Área de Vigilancia de Riesgos Ambientales en Salud, Dirección General de Salud Pública, Consejería de Sanidad, Comunidad de Madrid; 3 Departamento de Zoología y Antropología Física, Facultad de Ciencias Biológicas, Universidad Complutense de Madrid; Liverpool School of Tropical Medicine, UNITED KINGDOM

## Abstract

**Background:**

An outbreak of human leishmaniasis due to *Leishmania infantum* has been registered in an urban area of southwestern Madrid, Spain, since 2010. Entomological surveys carried out in the municipalities of Fuenlabrada, Leganés, Getafe and Humanes de Madrid showed that *Phlebotomus perniciosus* is the only potential vector. In this work, an intensive molecular surveillance was performed in *P*. *perniciosus* females captured in the region between 2012 and 2018.

**Methodology/Principal findings:**

A total of 1805 *P*. *perniciosus* females were analyzed for *Leishmania* infection, and 1189 of them also for bloodmeal identification. Eleven different species of vertebrate were detected by amplification and subsequent sequencing of the 359 bp cyt*b* fragment. The most prevalent blood source identified was hare (n = 553, 46.51%), followed by rabbit (n = 262, 21.95%). Less frequent were cat (n = 45, 3.80%), human (n = 34, 2.90%), pig (n = 14, 1.20%), horse (n = 11, 0.93%), sheep (n = 3, 0.25%), rhea (n = 3, 0.25%), partridge (n = 1, 0.09%) and chicken (n = 1, 0.09%). The distribution of the blood meal sources varied between the different locations. Regarding *L*. *infantum* detection, PCR amplification of a fragment of kDNA, *cpb* gene and ITS1 region showed 162 positive specimens (8.97%). The highest infection rate was found in the municipality of Leganés (15.17%).

**Conclusions:**

The results of this molecular survey in *P*. *perniciosus*, the only leishmaniasis vector in the outbreak occurred in southwestern Madrid region, showed its opportunistic blood-feeding behaviour, high infection rates and the differences between the different points. This study was an essential part of the intensive surveillance plan in the area and the results obtained have supported the implementation of control measures in the outbreak.

## Author summary

Vector-borne diseases (VBD) comprise a pathogen-vector-reservoir relation, and control programs should take into account different factors involving this relationship. This study is focused on a leishmaniasis outbreak that has occurred in the southwestern Madrid region since 2010. An intensive molecular surveillance of the sand fly vector *Phlebotomus perniciosus* was carried out from 2012 to 2018 in 4 municipalities from the affected region: Fuenlabrada, Leganés, Getafe and Humanes de Madrid. The aim of this investigation was to discern how much the vector was infected and find out its blood-feeding habits in the different surveyed points. The results showed an important rate of infected sand flies and also that *P*. *perniciosus* presents an opportunistic blood-feeding behaviour, taking blood from many different hosts, depending on their availability and density. The results obtained in this surveillance helped the authorities to plan and implement more accurate and effective control measures in the area, reflecting the importance of applying molecular surveillance in VBD outbreaks.

## Introduction

Leishmaniasis is a vector-borne disease transmitted by female sand flies that causes between 70,000 and 1 million new cases annually and with about 1 billion people at risk of infection according to the last reports from the WHO [1]. This infectious disease is caused by flagellates from different *Leishmania* species that can cause different forms of leishmaniasis, from self-limited cutaneous injuries to the more severe visceral form [2]. Leishmaniasis is present in 98 countries, hitting mostly low-income populations. An expansion of the disease is linked to environmental changes due not only to climate change but also to deforestation, irrigation schemes or urbanization [3].

The control of vector-borne diseases (VBD) comprises numerous types of measures that involve the control population of the vector and potential reservoirs. The design and further implementation of control programs need to be supported by an excellent knowledge of the epidemiology of the disease and the biology of the vectors and reservoirs involved [4].

In Spain, more than 8,000 hospitalizations related to leishmaniasis have been registered between 1997 and 2011 [5]. The only species present, *Leishmania infantum*, is hypoendemic and causes both cutaneous and visceral leishmaniasis (CL and VL) mainly in the Mediterranean basin and the center of the country. In 2009, a significant increase of cases was identified in southwestern of the Community of Madrid. Until June 2020, 479 cases of CL and 303 of VL have been registered, with a peak of 197 cases in 2011 (data provided by Community of Madrid). The research carried out in the region pointed that this increase of leishmaniasis cases could be related to environmental changes that occurred in the area, where a large green area was constructed in a former agricultural land, leading to the isolation and increase of wild animals that could act as reservoirs and support the increase of sand fly population [6,7]. Further epidemiological studies found a correlation between the cases and the location, finding a concentration of cases close to the green area [7]. The significant population of hares and rabbits observed in this green area make them suspects of being potential reservoirs of the parasite and further xenodiagnosis experiments revealed their role as active hosts of *L*. *infantum* in the outbreak [8,9]. Moreover, molecular studies exposed significant infection rates among these lagomorphs [8–10]. In order to have a complete vision of the parasite cycle in the region, it was crucial to undertake entomological surveillance. Sand fly captures have been performed since 2012 to know sand fly population density in different points, sand fly dynamics, infection rates and bloodmeal preferences. All this information allowed the local and regional governments to implement different control programs to take over the situation, leading to the control of the number of cases [11].

In this study, we present the results from 7 years of molecular surveillance of female *P*. *perniciosus* sand flies captured in the area affected by a leishmaniasis outbreak in southwestern Madrid, Spain. A total of 1805 specimens were analyzed for *L*. *infantum* infection, showing high infection rates. Moreover, bloodmeal identification of 1189 engorged females was performed, revealing an opportunistic feeding behaviour of the vector in the different spatial areas of the outbreak area. This outcome was crucial for the implementation of control measures carried out by the corresponding authorities in Madrid and highlights the importance of molecular surveillance in areas affected by a vector-borne disease like leishmaniasis.

## Methods

### Survey collection

**Fig 1:**
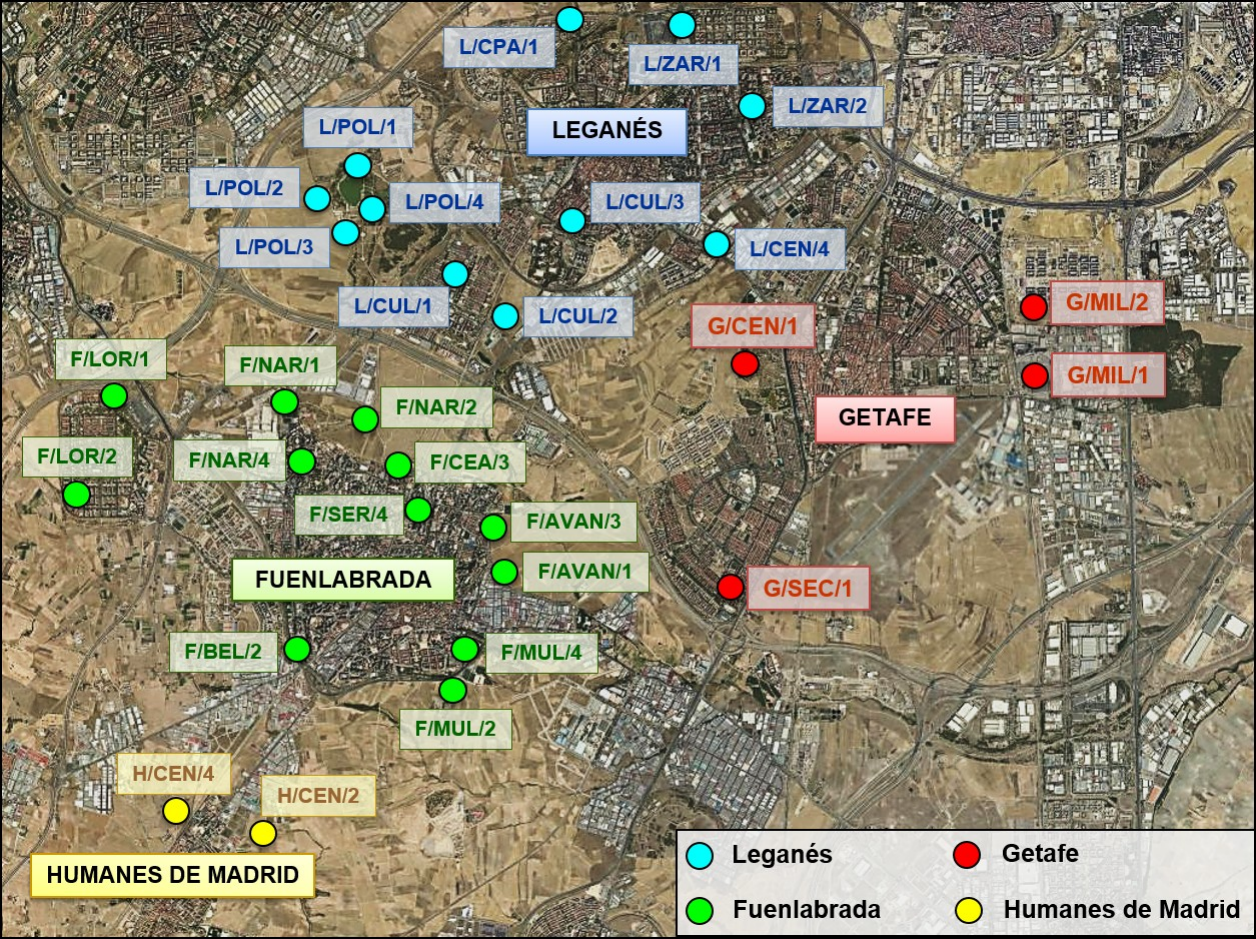
Location of collection points. The map (https://ngmdb.usgs.gov/mapview/?center=-3.791,40.291&zoom=13) shows the location of each collection site in the four municipalities surveyed.

The entomological survey was conducted in the municipalities affected by the human leishmaniasis outbreak in the southwestern region of Madrid: Fuenlabrada, Leganés, Getafe and Humanes de Madrid. The map (https://ngmdb.usgs.gov/mapview/?center=-3.791,40.291&zoom=13) shows sampling points involved urban and periurban sites as green parks, courtyards of public and private buildings located in the municipalities mentioned above (Fig 1, S1 Table).

Both sticky traps (ST) and CDC light traps (LT) were placed during the active season of the sand flies, from May to November, for 7 subsequent years (2012–2018). The traps were placed and collected by technical staff of the Community of Madrid. The distribution of the traps varied during the different epidemiological seasons in relation to the epidemiological data. Subsequently, the traps were examined for collecting the sand flies at the Facultad de Ciencias Biológicas (Universidad Complutense de Madrid). Sand flies were stored in ethanol 70% at 4°C for further taxonomic identification and molecular analysis that was carried out at the Laboratorio de Entomología Médica (Instituto de Salud Carlos III). The taxonomic identification procedure involved the separation of the head and genitalia of the samples that were mounted and observed under the microscope. On the other hand, the thorax and the abdomen were used for DNA extraction following the procedure described by Jiménez et al., 2013 [12]. During the dissection step, blood digestion degree was observed and noted following the scale described by Dolmatova & Demina, 1971 [13]. DNA samples were stored at -20°C until molecular analysis.

### Identification of blood meal sources

Blood meal sources of engorged sand fly females were identified by the amplification and further sequencing of a fragment of 359 bp of vertebrate cytochrome *b* (cyt *b*) gene as described in Jimenez et al., 2013 [12]. Degenerated primers were used to analyze the samples: cyt_bb1 (5´-CCA TCM AAC ATY TCA DCA TGA TGA AA-3´) and cyt_bb2 (5´-GCH CCT CAG AAT GAY ATT TGK CCT CA-3) [14]. PCR amplification was carried out loading 40ng of DNA in a final volume of 25 μl: 1x Buffer (Biotools B&M Labs, Spain), 1.5 mM MgCl2 (Biotools B&M Labs, Spain), 100 μM dNTPs mixture (Biotools B&M Labs, Spain), 5U of *Hotsplit* polymerase (Biotools B&M Labs, Spain), 1 μl of BSA DNAse Free (20 mg/ml, Roche, Basel, Switzerland) and 0.1 μl of each primer. PCR conditions were: one cycle of 6 minutes at 94°C, 40 cycles consisting of denaturation at 94°C for 30 seconds, annealing at 55°C for 30 seconds and elongation at 72°C for 45 seconds; final elongation was at 72°C for 10 minutes.

PCR products obtained were separated on a 1.5% agarose gel (Conda, Spain) stained with “Pronasafe Nucleic Acid Staining Solution” (10 mg/ml) (Conda, Spain) and visualized under UV light. The bands visualized were removed from the gel under UV exposure and purified using the SpeedTools PCR Clean-up kit (Biotools B&M Labs, Spain). Sequencing was carried out in an ABI PRISM 3730XL DNA Analyzer (Applied Biosystems, EEUU) and the subsequent electropherograms were manually inspected and corrected using ChromasPro program (McCarthy, Queensland, Australia). Finally, to identify the obtained nucleotide fragments, homologies with the available sequences data in GenBank were carried out with the software BLAST (http://www.ncbi.nlm.nih.gov/BLAST).

### *Leishmania infantum* detection

*P*. *perniciosus* females were examined for the detection of *Leishmania* spp. by the amplification of a 120 bp fragment from kinetoplast DNA (kDNA) and further analysis by cysteine proteinase *b* (*cpb*) following protocols previously described [12]. Those samples positive to one of the previous reactions were submitted to the amplification of the internal transcribed region 1 (ITS1) PCR following the protocols described by El Tai et al. 2000 with few modifications to adapt it to the reagents used in our laboratory: 1x Buffer (Biotools B&M Labs, Spain), 1.5 mM MgCl2 (Biotools B&M Labs, Spain), 100 μM dNTPs mixture (Biotools B&M Labs, Spain), 5U of *Hotsplit* polymerase (Biotools B&M Labs, Spain), 1 μl of BSA DNAse Free (20 mg/ml, Roche, Basel, Switzerland) and 25 pmol of each primer. PCR conditions were: one cycle of 6 minutes at 94°C, 32 cycles consisting of denaturation at 95°C for 20 seconds, annealing at 53°C for 30 seconds and elongation at 72°C for 1 minute; final elongation was at 72°C for 10 minutes [15,16]. Electrophoresis of the PCR products was carried out as explained in the previous section. In the case of ITS1 bands obtained, they were excised from the gel and purified for further sequencing and identification by homology search using BLAST (http://www.ncbi.nlm.nih.gov/BLAST) as previously described.

## Results

### Sand fly collection

A total of 1805 *P*. *perniciosus* female sand flies were analyzed. Regarding the type of collection, 1211 of the females were captured by ST, while the rest (n = 594) were collected using LT. More than half of the samples were captured in Leganés (n = 896), followed by Fuenlabrada (n = 570), Getafe (n = 327) and Humanes de Madrid (n = 12). From the 1805 collected females, 1189 were blood-engorged while 616 did not present ingested blood in the midgut.

### Blood meal identification

Blood meal identification was achieved in 941 blood fed *P*. *perniciosus* females meaning an efficiency of 79.10%. Eleven different species of vertebrates were detected by amplification and subsequent sequencing of the 359 bp cyt*b* fragment. The most prevalent blood source identified was hare (n = 553, 46.51%), followed by rabbit (n = 262, 21.95%), cat (n = 45, 3.80%), human (n = 34, 2.90%), pig (n = 14, 1.20%) and horse (n = 11, 0.93%). DNA from other animals was found in a less frequency: sheep (n = 3, 0.25%), rhea (n = 3, 0.25%), partridge (n = 1, 0.09%) and chicken (n = 1, 0.09%). Moreover, fourteen mixed blood meal sources were identified by electropherogram analysis: hare/rabbit (n = 5, 0.51%), hare/human (n = 2, 0.17%), cat/rabbit (n = 1, 0.09%), rabbit/turkey (n = 1, 0.09%), chicken/pig (n = 1, 0.09%). We could even differentiate between two cyt*b* hare haplotypes, Iberian hare (*Lepus granatensis*) and mountain hare (*Lepus timidus*) in four female sand flies (S1 Fig). We were not able to identify the blood source from 248 out of 1189 the samples (20.90%).

**Fig 2:**
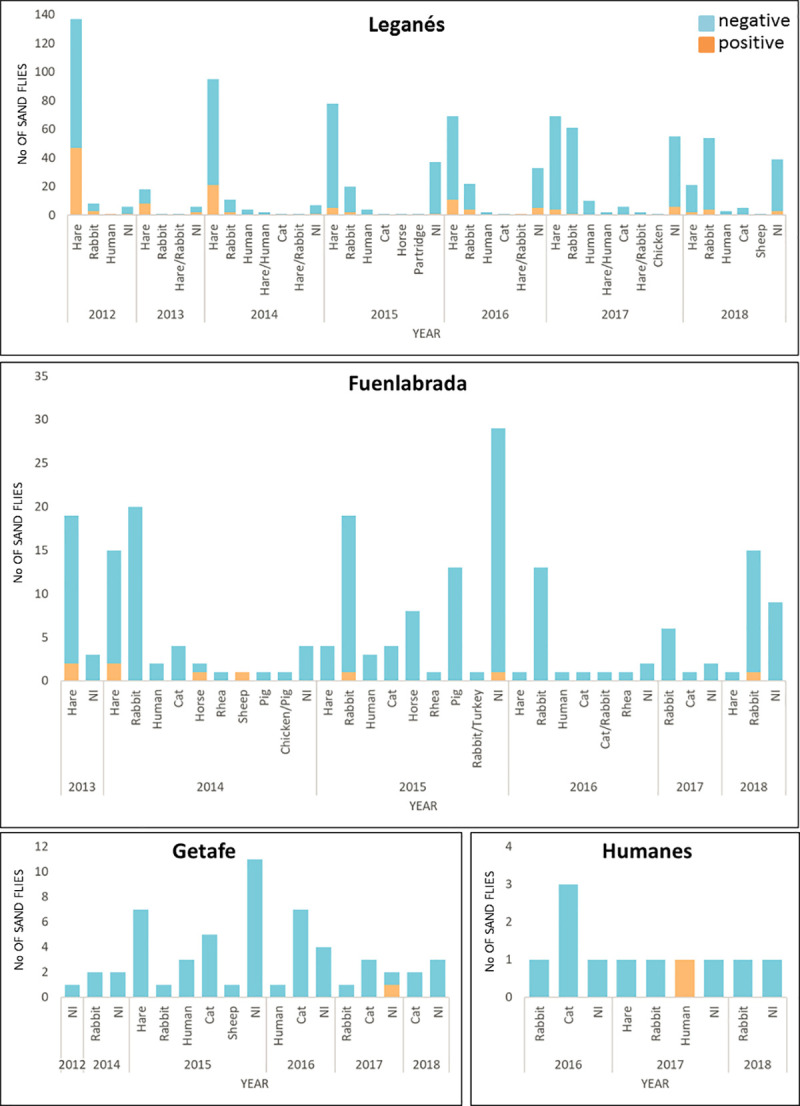
Bloodmeals preferences in the surveyed municipalities from 2012 to 2018. The bloodmeal identification showed a higher variety of bloodmeals sources in Fuenlabrada and Leganés, with 10 and 8 different sources, respectively. The number of positive and negative samples is shown in S2 Table.

The distribution of the blood meal sources varied between the different locations, as showed in Fig 2. Fuenlabrada presented the highest diversity of blood sources (n = 10) followed by Leganés (n = 8), Getafe (n = 5) and Humanes de Madrid (n = 5). In Leganés, the number of sand flies that presented hare blood declines from 2012 to 2018 from 137 to 19, while sand flies fed on rabbit increase from 8 to 54. In the other hand, in Fuenlabrada, hare as blood source also decreases along the survey, while rabbit decreases from 2012 to 2017 but shows a slight increase the last year. In Getafe and Humanes de Madrid, the number of engorged sand flies is smaller as well as the variety of animals the sand flies fed on during the yearly surveys.

### *Leishmania infantum* detection

PCR amplification for *Leishmania* detection in the *P*. *perniciosus* females showed 162 positive specimens (8.97%). Sequencing results of these ITS1 products showed that all positive sand flies were infected by *L*. *infantum*.

Regarding the different locations, the highest infection rate was found in Leganés (15.17%), followed by Humanes de Madrid (8.33%), Fuenlabrada (3.50%) and Getafe (1.52%) (Table 1). From the 162 positive sand flies, 16 of them were unfed (9.88%).

**Table 1:** Number of *Phlebotomus perniciosus* analyzed from each location annually.

Municipality	Year							
	2012	2013	2014	2015	2016	2017	2018	Total
Leganés	152 (45)	26 (10)	120 (31)	142 (8)	130 (22)	208 (11)	118 (9)	896 (136)
Fuenlabrada		22 (2)	284 (15)	210 (2)	20 (0)	9 (0)	25 (1)	570 (20)
Getafe	1 (0)		83 (3)	219 (1)	12 (0)	7 (1)	5 (0)	327 (5)
Humanes de Madrid			1 (0)		5 (0)	4 (1)	2 (0)	12 (1)
Total	153 (45)	48 (12)	488 (49)	571 (11)	167 (22)	228 (13)	150 (10)	1805 (162)

The number of sand flies positive to *Leishmania infantum* in each location and year is indicated in brackets.

Table 2 shows the number of positive sand flies regarding the blood source detected. Most of the females positive to *L*. *infantum* presented hare blood in their midguts (62.96%), meaning that 102 sand flies of a total of 553 (18.44%) fed on hares that were infected. From the sand flies fed on sheep, 1 out of 3 was infected (33.3%). *L*. *infantum* DNA was also found in females that presented blood from rabbit, human, horse and mixed hare/rabbit. No positive samples were detected in those engorged *P*. *perniciosus* females fed on cat, pig, rhea, partridge, chicken and the mixed bloodmeals hare/human, cat/rabbit, rabbit/turkey, chicken/pig. The distribution of bloodmeal sources and samples positive to *L*. *infantum* is shown in Fig 2. In Leganés and Fuenlabrada, where most of the samples were collected, the number of positive engorged females decreases along the years.

**Table 2:** Relation of positive sand flies regarding their blood meal source.

Blood meal source	N*o P*. *perniciosus* positive to *L*. *infantum* (%)
Hare	102 (62.96)
Rabbit	19 (11.73)
Human	2 (1.23)
Horse	1 (0.62)
Sheep	1 (0.62)
Hare/Rabbit	1 (0.62)
Unknown	20 (12.35)
Unfed	16 (9.88)

## Discussion

This study shows a molecular follow-up of 1805 *P*. *perniciosus* females collected across an extensive surveillance in the most important human leishmaniasis outbreak in Europe occurred in Madrid, Spain. Here, we recapitulate the data obtained from 2012–2018 focusing on feeding habits and *L*. *infantum* infection rates. To our knowledge, this is the largest molecular survey for sand fly bloodmeal identification in a leishmaniasis outbreak.

Blood meal sources from the engorged female sand flies were analyzed by cyt*b* amplification and further sequencing of the fragments with high efficiency (79.1%). The blood source of 248 females could not be identified. In this sense, multiple factors can affect this analysis, such as the presence of inhibitors in blood, the high content of proteins present in the chitinous cuticle of insects, the amount of blood present and its degree of digestion [16]. The percentage of identified blood meal sources in this study is similar to the reported in other studies [17–19]. The analysis of these fragments by BLAST showed a wide variety of feeding sources. These results matched with the opportunistic behaviour previously described in *P*. *perniciosus* in the same area, as well as in other regions [17,20–22]. Specifically in this survey, one of the collection points was located close to a farm-school in the municipality of Fuenlabrada (F/BEL/2 in Fig 1), which presented the highest blood meal diversity with 9 different blood sources. The finding of DNA of uncommon animals like turkey, partridge or rhea between the blood meals supports the opportunistic behaviour of this dipteran. In the case of rhea’s blood, we know that is one of the animals present in the farm-school located in Fuenlabrada, as well as horses, pigs or chickens. Regarding partridge blood, although in low numbers, this bird is also one of the wild animals present in the parks that surround Fuenlabrada and Leganés. Studies carried out in a wildlife park, and zoos have also described a wide range of bloodmeals due to the high variability of animals in such areas [23]. However, Pérez-Cutillas et al. 2020 found that *P*. *perniciosus*, *P*. *ariasi* and *P*. *papatasi* under similar circumstances showed a preference for feeding on certain animals in the studied wildlife park. Specifically, they describe that although there was a higher percentage of sand flies fed on fallow deer (*Dama dama*) and red deer (*Cervus elaphus*), the study after considering movement cost predicted the highest probability of feeding on red deer and common eland (*Taurotragus oryx*), with a positive association with host census [24]. Those findings would support that, despite the wide range of blood sources available, sand flies in our study prefer to feed on the blood of those that were most available, such as hares (46.51%) and rabbits (21.95%). Both leporids have an important presence in the region, and previous studies of xenodiagnoses have described their essential role in the outbreak as potential reservoirs of the parasite [8,9]. In this sense, the Community of Madrid implemented measures to control the populations of hares, mainly by catching the animals by using nets, and also rabbits [11]. The decrease of sand flies fed on hares from 2012 is very clear, showing that the measures taken were effective. However, this reduction is not so clear in case of sand flies fed on rabbit. This difference could be due to the fact that the capture of rabbits is more difficult as well as the rabbits could proliferate in those areas where the hares were eliminated [11].

In Spain, the dog is suggested as the main reservoir of *L*. *infantum*. However, we did not find any *P*. *perniciosus* female that fed on this animal. Similar results were described in another study carried out in the area in which dog blood detected in only 0.33% of 912 *P*. *perniciosus* examined [21]. Although it could be surprising, prevalence studies on dogs of the affected region have shown that canine leishmaniasis has not increased [25], probably due to the measures that owners take to protect dogs like the use of collars or pipettes [26].

Regarding the considerable number of people affected in the outbreak, close to 800, it would be expected to find that a significant number of sand flies fed on humans. However, only 35 females out of 1189 presented human blood in their midguts. One reason for this finding would be that the majority of collection points were placed in parks and green areas, where other animals were much more accessible and abundant than humans, acting then as accidental hosts.

On the other hand, the study of the feeding habits also highlights the difference between the four surveyed municipalities. The most notorious difference is the higher number of females feeding on hares in Leganés in comparison with the other three locations. The population density of hares is also higher in this municipality [27]. In any case, these results support other studies carried out in the area and the hypothesis of the influence of physical barriers as roads and railways [21].

Identification of blood-sucking food sources of insects that transmit pathogens, such as sand flies, could reveal potential reservoirs. This data is essential when it comes to knowing the transmission cycle and the development of effective control strategies. Likewise, identifying food preferences allows the determination of the contact between the vector and the susceptible hosts in a given region or area, revealing the risk of exposure [28,29]. The high number of sand flies feeding on hares, and rabbits found in the present study support the role of these lagomorphs as the main reservoirs in the outbreak as previous studies revealed [8,9]. For the rest of the animals detected, some have been confirmed as reservoirs of the parasite and sand flies are able to acquire the pathogen during the blood intake, like cats and humans [30,31]. In this case, they can maybe play the role of secondary reservoirs in the area. Those animals that have not been confirmed as reservoirs, like horse and sheep, can be simple blood sources that allow the maintenance of *P*. *perniciosus* population in the area [32–36].

The *L*. *infantum* infection average rate detected in this study was 8.97%, although it reached 15.17% in the municipality of Leganés. At the same time, Leganés presented the highest number of females fed on hare, which is the primary blood source detected in infected sand flies. Hence, the relation of the infection rate and blood meal source could explain these results, and, as mention previously, would support the critical role of hares in the life cycle of the parasite in the region. The infection rates found in the present study are very similar to the results described in a different intensive survey in four sampling stations that took place between 2012–2014 in the same region, which described an average infected rate of 13.31% and 7.78% in blood-fed and unfed *P*. *perniciosus* females, respectively [21]. However, other studies carried out in different areas of the Iberian Peninsula reported lower infection rates [17,18].

In conclusion, the analysis of 606 unfed and 1189 engorged *P*. *perniciosus* females, following a leishmaniasis outbreak in Madrid, Spain, showed high *L*. *infantum* infection rates and a remarkable opportunistic blood-feeding behaviour on this sand fly species. The results of this study show the evolution of the blood meal sources and infection rates in the mentioned outbreak and the effect of the control measures taken by the Community of Madrid authorities. In this respect, this molecular monitoring study showed to be an essential part of the surveillance plan in the area and the results obtained from these surveys have supported the implementation of control measures in the outbreak and helped in the understanding of the *Leishmania* transmission cycle in this periurban leishmania outbreak.

## Supporting information

S1 Table Trap codes, municipality and geolocation of the collection points. (DOCX)

S2 Table Bloodmeal preferences in each municipality along the different years of surveillance (2012–2018) and number of positive samples to *Leishmania infantum* by PCR. (DOCX)

S1 Fig Electropherograms of the mixed bloodmeals detected. The presence of different blood sources in the *Phlebotomus perniciosus* female midguts was identified by the double peaks in the electropherograms after sequencing the 359 bp cyt*b* fragment. Double peaks are highlighted by orange dots.
(TIF)
