# Supplementary material for: Opportunistic feeding behaviour and Leishmania infantum detection in Phlebotomus perniciosus females collected in the human leishmaniasis focus of Madrid, Spain (2012–2018)
Source: PLoS Negl Trop Dis. 2021 Mar 15;15(3):e0009240. doi: 10.1371/journal.pntd.0009240 (PMC7993803; doi:10.1371/journal.pntd.0009240)
Supplement: S1 Table — (DOCX) [file pntd.0009240.s001.docx]

| Code | **Location** | **Geolocation** | |
| --- | --- | --- | --- |
|  |  | Latitude | Longitude |
| F/AVAN/1 | Fuenlabrada | 40.294807 | -3.776533 |
| F/AVAN/3 | Fuenlabrada | 40.297636 | -3.781895 |
| F/MUL/2 | Fuenlabrada | 40.276893 | -3.790408 |
| F/MUL/4 | Fuenlabrada | 40.280855 | -3.790689 |
| F/SER/4 | Fuenlabrada | 40.290531 | -3.793237 |
| F/BEL/2 | Fuenlabrada | 40.290489 | -3.809766 |
| F/CEA/3 | Fuenlabrada | 40.300506 | -3.802143 |
| F/NAR/1 | Fuenlabrada | 40.304951 | -3.810704 |
| F/NAR/2 | Fuenlabrada | 40.305173 | -3.806047 |
| F/NAR/4 | Fuenlabrada | 40.298765 | -3.805112 |
| F/LOR/1 | Fuenlabrada | 40.308922 | -3.832593 |
| F/LOR/2 | Fuenlabrada | 40.308922 | -3.832593 |
| H/CEN/1 | Humanes de Madrid | 40.247941 | -3.820946 |
| H/CEN/3 | Humanes de Madrid | 40.259334 | -3.82654 |
| G/CEN/1 | Getafe | 40.307136 | -3.745748 |
| G/MIL/1 | Getafe | 40.315326 | -3.706952 |
| G/MIL/2 | Getafe | 40.312687 | -3.711126 |
| G/SEC/1 | Getafe | 40.286216 | -3.742989 |
| L/CEN/4 | Leganés | 40.323482 | -3.761865 |
| L/CPA/1 | Leganés | 40.343503 | -3.762726 |
| L/CUL/1 | Leganés | 40.317751 | -3.792755 |
| L/CUL/2 | Leganés | 40.31216 | -3.784334 |
| L/CUL/3 | Leganés | 40.319374 | -3.779368 |
| L/POL/1 | Leganés | 40.32679 | -3.795027 |
| L/POL/2 | Leganés | 40.32447 | -3.798401 |
| L/POL/3 | Leganés | 40.322655 | -3.797662 |
| L/POL/4 | Leganés | 40.32493 | -3.790367 |
| L/ZAR/1 | Leganés | 40.342721 | -3.751473 |
| L/ZAR/4 | Leganés | 40.326968 | -3.753676 |

**S1 Table**. **Trap codes, municipality and geolocation of the collection points.**
