# Supplementary material for: Opportunistic feeding behaviour and Leishmania infantum detection in Phlebotomus perniciosus females collected in the human leishmaniasis focus of Madrid, Spain (2012–2018)
Source: PLoS Negl Trop Dis. 2021 Mar 15;15(3):e0009240. doi: 10.1371/journal.pntd.0009240 (PMC7993803; doi:10.1371/journal.pntd.0009240)
Supplement: S2 Table — (DOCX) [file pntd.0009240.s002.docx]

| LEGANÉS | | | |
| --- | --- | --- | --- |
|  |  | Positive | Negative |
| 2012 | Hare | 47 | 90 |
|  | Rabbit | 3 | 5 |
|  | Human | 1 | 0 |
|  | NI | 1 | 5 |
| 2013 | Hare | 8 | 10 |
|  | Rabbit | 0 | 1 |
|  | Hare/Rabbit | 0 | 1 |
|  | NI | 2 | 4 |
| 2014 | Hare | 21 | 74 |
|  | Rabbit | 2 | 9 |
|  | Human | 0 | 4 |
|  | Hare/Human | 0 | 2 |
|  | Cat | 0 | 1 |
|  | Hare/Rabbit | 0 | 1 |
|  | NI | 1 | 6 |
| 2015 | Hare | 5 | 73 |
|  | Rabbit | 2 | 18 |
|  | Human | 0 | 4 |
|  | Cat | 0 | 1 |
|  | Horse | 0 | 1 |
|  | Partridge | 0 | 1 |
|  | NI | 1 | 36 |
| 2016 | Hare | 11 | 58 |
|  | Rabbit | 4 | 18 |
|  | Human | 0 | 2 |
|  | Cat | 0 | 1 |
|  | Hare/Rabbit | 1 | 0 |
|  | NI | 5 | 28 |
| 2017 | Hare | 4 | 65 |
|  | Rabbit | 1 | 60 |
|  | Human | 0 | 10 |
|  | Hare/Human | 0 | 2 |
|  | Cat | 0 | 6 |
|  | Hare/Rabbit | 0 | 2 |
|  | Chicken | 0 | 1 |
|  | NI | 6 | 49 |
| 2018 | Hare | 2 | 19 |
|  | Rabbit | 4 | 50 |
|  | Human | 0 | 3 |
|  | Cat | 0 | 5 |
|  | Sheep | 0 | 1 |
|  | NI | 3 | 36 |

**S2 Table**. **Bloodmeal preferences in each municipality along the different years of surveillance (2012-2018) and number of positive samples to *Leishmania infantum* by PCR.**

| FUENLABRADA | | | |
| --- | --- | --- | --- |
|  |  | Positive | Negative |
| 2013 | Hare | 2 | 17 |
|  | NI | 0 | 3 |
| 2014 | Hare | 2 | 13 |
|  | Rabbit | 0 | 20 |
|  | Human | 0 | 2 |
|  | Cat | 0 | 4 |
|  | Horse | 1 | 1 |
|  | Rhea | 0 | 1 |
|  | Sheep | 1 | 0 |
|  | Pig | 0 | 1 |
|  | Chicken/Pig | 0 | 1 |
|  | NI | 0 | 4 |
| 2015 | Hare | 0 | 4 |
|  | Rabbit | 1 | 18 |
|  | Human | 0 | 3 |
|  | Cat | 0 | 4 |
|  | Horse | 0 | 8 |
|  | Rhea | 0 | 1 |
|  | Pig | 0 | 13 |
|  | Rabbit/Turkey | 0 | 1 |
|  | NI | 1 | 28 |
| 2016 | Hare | 0 | 1 |
|  | Rabbit | 0 | 13 |
|  | Human | 0 | 1 |
|  | Cat | 0 | 1 |
|  | Cat/Rabbit | 0 | 1 |
|  | Rhea | 0 | 1 |
|  | NI | 0 | 2 |
| 2017 | Rabbit | 0 | 6 |
|  | Cat | 0 | 1 |
|  | NI | 0 | 2 |
| 2018 | Hare | 0 | 1 |
|  | Rabbit | 1 | 14 |
|  | NI | 0 | 9 |

| GETAFE | | | |
| --- | --- | --- | --- |
|  |  | Positive | Negative |
| 2012 | NI | 0 | 1 |
| 2014 | Rabbit | 0 | 2 |
|  | NI | 0 | 2 |
| 2015 | Hare | 0 | 7 |
|  | Rabbit | 0 | 1 |
|  | Human | 0 | 3 |
|  | Cat | 0 | 5 |
|  | Sheep | 0 | 1 |
|  | NI | 0 | 11 |
| 2016 | Human | 0 | 1 |
|  | Cat | 0 | 7 |
|  | NI | 0 | 4 |
| 2017 | Rabbit | 0 | 1 |
|  | Cat | 0 | 3 |
|  | NI | 1 | 1 |
| 2018 | Cat | 0 | 2 |
|  | NI | 0 | 3 |

| HUMANES de MADRID | | | |
| --- | --- | --- | --- |
|  |  | Positive | Negative |
| 2016 | Rabbit | 0 | 1 |
|  | Cat | 0 | 3 |
|  | NI | 0 | 1 |
| 2017 | Hare | 0 | 1 |
|  | Rabbit | 0 | 1 |
|  | Human | 1 | 0 |
|  | NI | 0 | 1 |
| 2018 | Rabbit | 0 | 1 |
|  | NI | 0 | 1 |
