# Supplementary figures and images for: Opportunistic feeding behaviour and Leishmania infantum detection in Phlebotomus perniciosus females collected in the human leishmaniasis focus of Madrid, Spain (2012–2018)
Source: PLoS Negl Trop Dis. 2021 Mar 15;15(3):e0009240. doi: 10.1371/journal.pntd.0009240 (PMC7993803; doi:10.1371/journal.pntd.0009240)

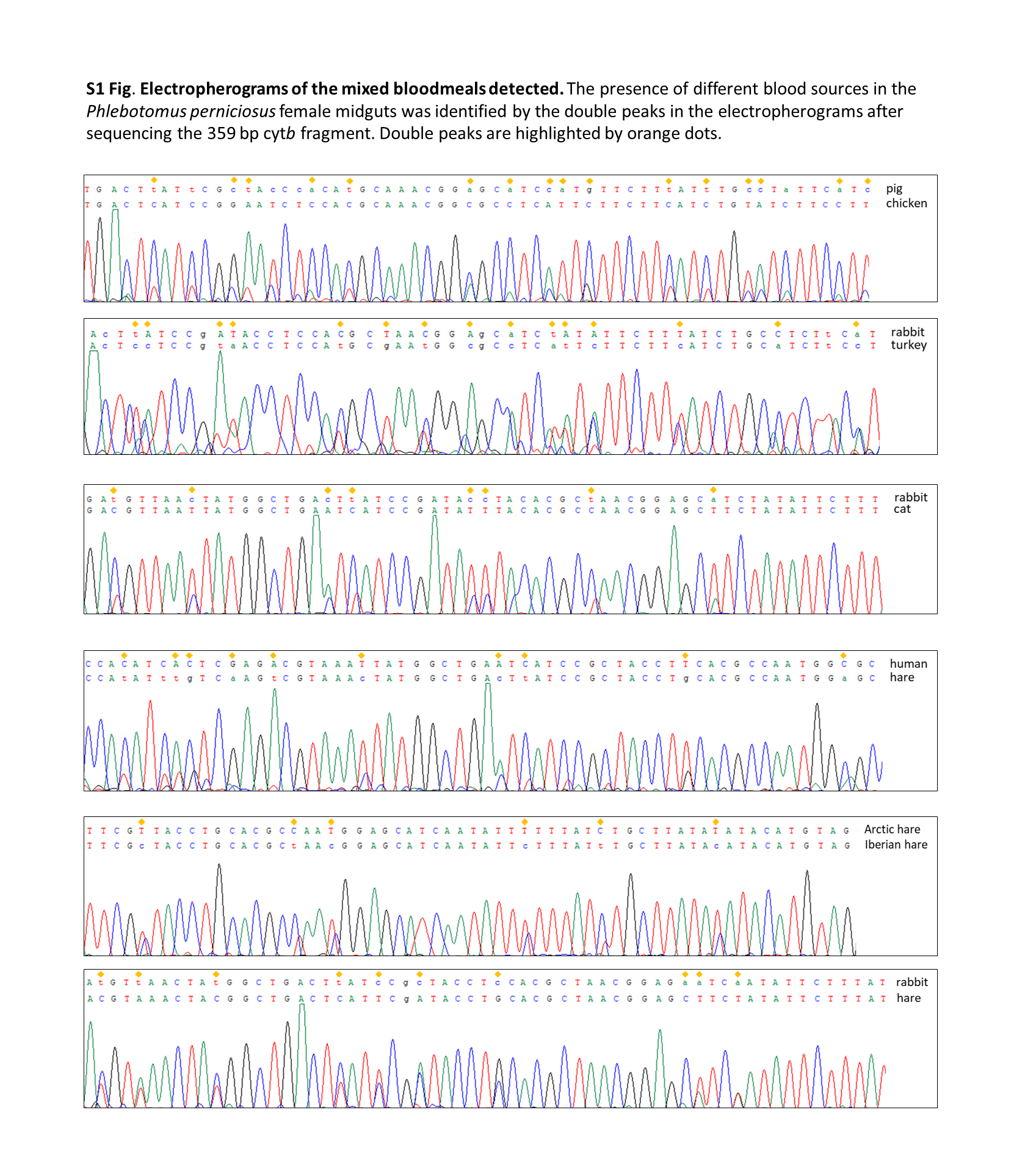

Supplement: S1 Fig — The presence of different blood sources in the Phlebotomus perniciosus female midguts was identified by the double peaks in the electropherograms after sequencing the 359 bp cytb fragment. Double peaks are highlighted by orange dots. (TIF) [file pntd.0009240.s003.tif]
